# Complications of oropharyngeal dysphagia in older individuals and patients with neurological disorders: insights from Mataró hospital, Catalonia, Spain

**DOI:** 10.3389/fneur.2024.1355199

**Published:** 2024-03-07

**Authors:** Tennekoon B. Karunaratne, Pere Clavé, Omar Ortega

**Affiliations:** ^1^Gastrointestinal Physiology Laboratory, Hospital de Mataró, Universitat Autònoma de Barcelona, Mataró, Spain; ^2^Centro de Investigación Biomédica en Red de Enfermedades Hepáticas y Digestivas (Ciberehd), Barcelona, Spain

**Keywords:** oropharyngeal dysphagia, swallowing disorders, aspiration pneumonia, clinical complications, post-stroke

## Abstract

**Background:**

Oropharyngeal dysphagia (OD) significantly impacts older individuals and neurologically compromised patients, hindering safe ingestion of food and liquids. Despite its prevalence, OD remains underdiagnosed and undertreated, leading to severe complications such as malnutrition, dehydration, respiratory infections, and aspiration pneumonia (AP), and increases hospital readmissions.

**Objectives:**

This study analyzes the intricate relationship between OD and various clinical complications in older individuals and patients with neurological disorders.

**Methods:**

Utilizing retrospective analysis and narrative review, our work consolidates findings from prior studies on Hospital de Mataro’s dysphagia patient cohort. Revisiting OD’s intricate association with clinical complications, it presents data via odds ratios (OR), incidence ratios (IR), and hazard ratios (HR) from univariate and multivariate analyses.

**Results:**

Five studies (2001–2014) involving 3,328 patients were scrutinized. OD exhibited independent and significant associations with various complications among older patients. Older individuals with OD faced heightened 1-month (ODDS 3.28) and 1-year (OR 3.42) mortality risks post-pneumonia diagnosis. OD correlated with a 2.72-fold risk of malnutrition, 2.39-fold risk of lower respiratory tract infections, 1.82-fold pneumonia readmissions (IR), and 5.07-fold AP readmissions (IR). Post-stroke OD is linked to neurological impairment (OR 3.38) and respiratory (OR 9.54) and urinary infections (OR 7.77), alongside extended hospital stays (beta coefficient 2.11).

**Conclusion:**

Oropharyngeal dysphagia causes and significantly exacerbates diverse clinical complications in older and post-stroke patients, emphasizing the urgent need for proactive identification, comprehensive assessment, and tailored management. Acknowledging OD’s broader implications in general medical practice is pivotal to improving patient outcomes and healthcare quality.

## Background and aims

Dysphagia encompasses a spectrum of conditions that includes both oropharyngeal and esophageal dysphagia. These two forms, while falling under the same umbrella term, have distinct characteristics and implications. However, the scope of this article is specifically confined to oropharyngeal dysphagia and any subsequent reference to “dysphagia” in this article should be understood as referring exclusively to oropharyngeal dysphagia, unless explicitly stated otherwise. Oropharyngeal dysphagia is a multifaceted medical condition with significant health implications for various patient groups. It emerges from complex interactions between neurology, gastroenterology, and related fields, extending beyond mere swallowing difficulty to present a broad challenge with wide-ranging clinical implications. Regrettably, dysphagia is often overlooked, misdiagnosed, or underestimated in general medical practice, leading to a series of clinical complications that demand immediate attention (1).

The reported prevalence of dysphagia in published literature varies greatly due to different diagnostic methods. Studies indicate that dysphagia affects a substantial portion of individuals aged 70 years or older, with estimates ranging from 27 to 91% (2, 3). For patients with neurological conditions, the prevalence can be as high as 50% (1, 3). This positions dysphagia as a key concern in geriatric care (4). Complications of dysphagia, such as aspiration pneumonia, are particularly worrisome. One study found that about 33% of older patients with dysphagia developed aspiration pneumonia during rehabilitation, while another reported a 48.2% prevalence in a cohort of patients with dysphagia (5, 6). Although estimates vary, it is generally recognized that individuals with dysphagia face a higher risk of aspiration pneumonia compared to those without swallowing difficulties. Cabre et al. (7) established a strong link between dysphagia and pneumonia in older patients, emphasizing the prognostic implications and increased risk of readmission due to dysphagia. Dysphagia is also closely associated with malnutrition, with studies indicating a significant prevalence ranging from 25 to 45% in various patient cohorts (8, 9). Those with dysphagia often struggle to consume food properly, leading to decreased nutrient intake and potential deficiencies in essential nutrients. This research has advanced our understanding of dysphagia as a precursor to malnutrition and respiratory tract infections, adding complexity to its management in independently-living older individuals.

Besides its impact on health and quality of life, untreated or poorly managed dysphagia and its complications present a significant economic burden (10–12). Research at Mataró Hospital found that post-stroke patients with dysphagia incurred notably higher costs at various stages than those without dysphagia during hospitalization (€5357.67 vs. €3976.30), 3 months post-hospitalization (€8242.0 vs. €5320.0), and 12 months’ follow up (€11,617.58 vs. €7242.78) (13). Importantly, patients with dysphagia who were at risk of malnutrition or malnourished and suffered respiratory infections incurred even higher costs at 12 months’ follow up (€19,817.58 vs. €7242.8) (13). These findings highlight the substantial financial impact of dysphagia, especially when coupled with complications, and emphasize the potential benefits of early intervention and effective management in reducing these costs while improving patient outcomes.

The wider significance of these findings is underscored in key reviews, leading to a broader understanding of dysphagia as a geriatric syndrome. Baijens et al.’s white paper, backed by the European Society for Swallowing Disorders and the European Union Geriatric Medicine Society, articulates this concept well (4). However, this research is just one aspect of a larger issue that requires collective recognition and action. Many caregivers, both informal and formal, may lack awareness and knowledge of dysphagia, resulting in less than optimal care practices (14–18). There is a pressing need for targeted education and training programs to enhance dysphagia recognition, safe feeding practices, and strategies for managing swallowing difficulties. While this remains a significant challenge, the use of artificial intelligence and innovative interventions like the Minimal-Massive Intervention offer a promising shift in dysphagia diagnosis and management (19, 20). Minimal-Massive Intervention employs advanced, less invasive, compensatory strategies, and oral health to achieve substantial therapeutic benefits with minimal physiological disruption. By providing caregivers with the necessary knowledge and skills, including awareness of innovative approaches like Minimal-Massive Intervention, we can optimize care for individuals with dysphagia and reduce associated risks and costs.

This narrative review highlights the crucial issue of limited awareness and understanding of dysphagia complications among caregivers, including general practitioners. Clinical complications resulting from delayed recognition and misdiagnosis can lead to malnutrition, aspiration pneumonia, and compromised quality of life. Through an in-depth review of comprehensive studies conducted at a single center, we aim to highlight the urgent need for proactive identification, tailored management, and increased awareness to enhance the diagnosis and management of dysphagia.

## Methods

In this review, we collected and compiled data from five separate investigations conducted by the same research team at Mataro Hospital between 2001 and 2014 (7, 20–23). These studies were carefully selected due to their shared location, consistent methodologies, and expert management. This selection aimed to ensure a uniform and cohesive research portfolio, enhancing the reliability of our findings for meaningful and comprehensive insights.

### Study selection and rationale

Each of the five studies was chosen based on their specific focus and shared attributes, ensuring a common purpose and execution. The key factors guiding their selection were:

1. Location and facility: all studies took place at Mataro Hospital conducted by the same research team, providing a standardized clinical and research environment for practices and methodologies.
2. Methodological consistency: a deliberate effort was made to select studies with consistent research methods, allowing for easier cross-referencing and comprehensive understanding.
3. Expert assessment: the studies were managed by well-recognized and experienced professionals with deep understanding of the subject matter, ensuring the reliability of the results.

### Methodological uniformity

Five studies examined dysphagia prevalence, risk factors, interventions, and complications in clinical settings like post-stroke, pneumonia, and older hospitalized populations. They all used the volume-viscosity swallow test, a validated tool for dysphagia diagnosis, assessing swallowing safety and efficacy with varying substance volumes and viscosities, and detecting silent aspirations via pulse-oximetry. Comprehensive evaluations were conducted on factors such as comorbidities (Charlson Comorbidity Index), functional abilities (Barthel Index), and nutritional status (Mini-Nutritional Assessment short-form). This uniformity allowed for a seamless approach within each study, contributing to coherence in the findings and enabling the research team to derive comprehensive conclusions from each individual study.

### Data extraction

Our data extraction process involved a detailed review of five studies conducted at Mataró Hospital, chosen for their shared location, consistent methods, and expert management. We have been using electronic medical records (TESIS/HCE; Nexus, Sabadell, Barcelona, Spain) since 2007, aiding in recording patient outcomes. This system facilitated the extraction process, which examined clinical databases and electronic notes for 3,328 patients in our dysphagia cohort. The researchers conducting the extraction were experienced and knowledgeable, ensuring the accuracy of the information. The data included variables like demographic details, clinical assessments, diagnostic tools like the volume-viscosity swallow test and videofluoroscopy, and outcome measures such as hospital readmissions and mortality rates. This detailed extraction process enhances our methodology’s transparency and validates our study’s findings.

### Outcome measures

A core set of clinical outcomes, including hospital readmissions, lower respiratory tract infections, pneumonia, and 6-month mortality after discharge, were assessed consistently across all studies. This uniformity in outcome measures simplified the comparison of results and enabled the identification of overarching trends and patterns.

Outlined below are concise summaries of the five studies, each addressing specific aspects of the research question, collectively contributing to a comprehensive synthesis of findings:

- Study 1, a prospective cohort in an acute geriatric unit, focused on dysphagia in pneumonia patients aged over 70 (134 patients; 80 female; mean age 78) (21). Dysphagia prevalence, clinical status, and prognosis were evaluated using bedside assessments, water swallow tests, and scoring systems like the Barthel Index, Mini Nutritional Assessment, Charlson Comorbidity Index, and Fine’s Pneumonia Severity Index. 55% showed dysphagia signs, correlating with older age, poorer functional status, higher comorbidity, and severe pneumonia. Dysphagia patients had higher 30-day (22.9 vs. 8.3%, *p* = 0.033) and 1-year mortality rates (55.4 vs. 26.7%, *p* = 0.001).
- Study 2 was a population-based cohort study, evaluating dysphagia as a risk factor for malnutrition and respiratory infections in 254 community-dwelling individuals aged 70 and over (118 female; mean age 78) with a 90% follow-up rate at 1-year (23). Dysphagia was assessed using the volume-viscosity swallow test, along with evaluations for malnutrition, hand grip, Barthel score, and lower respiratory tract infections. No significant difference in annual malnutrition risk was found between groups with or without dysphagia. However, prevalent malnutrition cases at follow-up were linked to baseline dysphagia and impaired swallow efficacy. Patients with impaired swallow safety had higher annual respiratory infection rates.
- Study 3 was an observational prospective cohort study, examining whether dysphagia is a determining factor for pneumonia-related readmissions in older patients discharged from an acute geriatric unit (7). Analyzing data from clinical databases and electronic notes of 2,359 patients (1,461 female, mean age 84.9 years) followed for an average of 24 months, the study found that dysphagia was diagnosed in 47.5% of cases. Individuals with dysphagia had a higher incidence rate of readmissions for pneumonia—6.7 readmissions per 100 person-years compared to 3.67 in those without dysphagia. Dysphagia correlated with a higher risk of hospitalization for pneumonia (hazard ratio 1.6), with significantly increased risks for both aspiration (hazard ratio 4.48) and non-aspiration pneumonia (hazard ratio 1.44).
- Study 4 was a prospective longitudinal study focusing on stroke patients admitted to a general hospital, investigating post-stroke dysphagia prevalence, related risk factors, and subsequent complications (22). Among the 395 stroke patients examined (184 female; mean age 73.2 years), a 45.06% prevalence of dysphagia was identified upon admission, with specific independently-associated risk factors including age, previous stroke history, stroke severity measured by the National Institute of Health Stroke Scale, and the volume of the lesion. Post-stroke dysphagia was independently linked to prolonged hospital stays, post-discharge institutionalization, diminished functional capacity, and notably higher mortality rates 3-month post-stroke. The study found that stroke severity and the patient’s status before the stroke played more pivotal roles in dysphagia development than the exact location of the lesion.
- Study 5 was an open-label trial of 186 hospitalized elderly patients with dysphagia (20). It evaluated the Minimal-Massive Intervention for reducing nutritional and respiratory complications in this population. 62 with dysphagia (29 female; mean age 84.8 years) received the Minimal-Massive Intervention, while an equivalent number formed the control group matched by sex, age, functionality, comorbidities, and body mass index followed standard clinical practices. Assessments included geriatric, comorbidity, functionality, frailty, oral dysphagia, nutritional status, and oral health measures. The Minimal-Massive Intervention encompassed fluid and food texture modifications, caloric and protein supplementation, and oral health guidance during and after hospitalization. The Minimal-Massive Intervention group showed significant improvements in nutritional status and functionality, lower hospital readmissions and respiratory infections, and higher 6-month survival rates than the control group.

### Statistical analysis

This study presents a comprehensive analysis of findings from five selected studies. Each study used univariate or multivariate analyses, calculating measures like odds ratios, incidence ratios, or hazard ratios to assess the associations between dysphagia and complications. Statistical methods included chi-square tests, Fisher exact tests, Mann–Whitney U tests, logistic regression, Cox regression, and survival analysis, with a consistent significance level set at *p* < 0.05. Our study synthesized these findings into a tabulated format, providing a consolidated overview of the associations between dysphagia and clinical outcomes, aiming to represent the statistical outcomes reported in the original studies comprehensively.

## Results

Five studies conducted between 2001 and 2014 were included in data compilation, comprising a cumulative cohort of 3,328 patients with dysphagia uniformly managed in the same institution. Our compilation in Table 1 summarized and presented the findings from these studies, revealing substantial associations between dysphagia and a range of clinical complications among older individuals and patients with neurological conditions.

**Table 1:** Summary of findings from five studies on dysphagia (2001–2014): this table compiles findings from five studies conducted at Mataro Hospital, all focusing on dysphagia.

Complications	Phenotype	ODDS/IR/HR	*p* value	Analysis	Reference
1-month mortality	Older with pneumonia	3.28 (1.13–9.50) (OR)	Significant	Univariate	Cabre et al. (21)
1-year mortality	Older with pneumonia	3.42 (1.64–7.11) (OR)	-	Univariate	Cabre et al. (21)
MN and risk of MN	Older from the community	2.72 (1.25–5.95) (ODDS)	0.010	Univariate	Serra-Prat et al. (23)
LRTI	Older from the community (with ISS)	2.39 (1.07–5.34) (OR)	0.030	Univariate	Serra-Prat et al. (23)
MN and risk of MN	Older from the community (with IES)	2.31 (0.96–5.57) (OR)	0.062	Multivariate (adjusted)	Serra-Prat et al. (23)
LRTI	Older from the community (with ISS)	2.55 (1.07–6.09) (OR)	0.035	Multivariate (adjusted)	Serra-Prat et al. (23)
Pneumonia readmissions	Older patients from AGU	1.82 (1.41–2.36) (IR)	-	Univariate	Cabre et al. (7)
Non-aspiration pneumonia readmissions	Older patients from AGU	1.37 (1.02–1.84) (IR)	-	Univariate	Cabre et al. (7)
Aspiration pneumonia readmissions	Older patients from AGU	5.07 (2.73–9.43) (IR)	-	Univariate	Cabre et al. (7)
Bronchoaspiration readmissions	Older patients from AGU	4.36 (2.91–6.52) (IR)	-	Univariate	Cabre et al. (7)
Pneumonia readmissions	Older patients from AGU	1.60 (1.15–2.20) (HR)	0.005	Multivariate	Cabre et al. (7)
Non-aspiration pneumonia readmissions	Older patients from AGU	1.44 (1.02–2.03) (HR)	0.037	Multivariate	Cabre et al. (7)
Aspiration pneumonia readmissions	Older patients from AGU	4.48 (2.01–10.0) (HR)	<0.001	Multivariate	Cabre et al. (7)
Bronchoaspiration readmissions	Older patients from AGU	3.02 (1.73–5.27) (HR)	<0.001	Multivariate	Cabre et al. (7)
Mortality	Older patients from AGU	1.82 (1.62–2.04) (HR)	<0.001	Multivariate	Cabre et al. (7)
Neurological complications	Post-stroke hospitalized	3.38 (1.58–7.25) (OR)	<0.001	Univariate	Rofes et al. (22)
Respiratory infections	Post-stroke hospitalized	9.54 (2.80–32.55) (OR)	<0.001	Univariate	Rofes et al. (22)
Urinary infections	Post-stroke hospitalized	7.77 (1.72–35.2) (OR)	<0.001	Univariate	Rofes et al. (22)
Hospital stay	Post-stroke hospitalized	2.11 (beta coefficient)	0.049	Univariate	Rofes et al. (22)
Mortality	Post-stroke hospitalized	27.34 (3.63–205.9) (OR)	<0.001	Univariate	Rofes et al. (22)
Respiratory infections	3-month post-stroke	4.87 (2.25–10.54) (OR)	<0.001	Univariate	Rofes et al. (22)
Mortality	3-month post-stroke	17.46 (5.39–56.51) (OR)	<0.001	Univariate	Rofes et al. (22)
Respiratory infections	12-month post-stroke	2.28 (1.35–3.85) (OR)	0.003	Univariate	Rofes et al. (22)
Mortality	12-month post-stroke	11.40 (5.19–25.04) (OR)	<0.001	Univariate	Rofes et al. (22)
Hospital stay	Post-stroke hospitalized	0.938 (beta coefficient)	0.049	Multivariate (adjusted)	Rofes et al. (22)
Mortality	3-month post-stroke	6.90 (1.57–30.34) (OR)	0.011	Multivariate (adjusted)	Rofes et al. (22)
General readmissions	Older hospitalized	2.78 (150–5.15) (IR)	0.001	Univariate	Martín et al. (20)
LRTI readmissions	Older hospitalized	5.97 (1.45–24.63) (IR)	0.002	Univariate	Martín et al. (20)
Readmissions for other causes (no LRTI/pneumonia)	Older hospitalized	2.79 (1.21–6.44) (IR)	0.011	Univariate	Martín et al. (20)

Key variables include comorbidities (Charlson Comorbidity Index), functional abilities (Barthel Index), and nutritional status (MNA short-form). The studies used uniform statistical methods to calculate measures such as Odds ratio (OR), Incidence ratio (IR), and Hazard ratio (HR), assessing the associations between dysphagia and its complications. All studies consistently set the significance level at *p* < 0.05. MN, Malnutrition; LRTI, Lower respiratory tract infection; ISS, Impaired safety of swallow; IES, Impaired efficacy of swallow; AGU, Acute geriatric unit; OR, Odds ratio; IR, Incidence ratio; and HR, Hazard ratio.

The data synthesis from these studies highlighted significant relationships between dysphagia and various clinical outcomes. For instance, older individuals with dysphagia and pneumonia exhibited a significant increase in 1-month mortality (odds ratio: 3.28, 95% CI: 1.13–9.50, *p* < 0.05), emphasizing the impact of pneumonia on short-term survival rates. Similarly, there was a substantial rise in 1-year mortality among this population (odds ratio: 3.42, 95% CI: 1.64–7.11, *p* < 0.05), underscoring the long-term consequences of pneumonia.

Additionally, our compilation emphasized the association between dysphagia and subsequent risks, such as malnutrition among older individuals from the community (odds ratio: 2.72, 95% CI: 1.25–5.95, *p* < 0.05), highlighting the importance of early nutritional evaluation and intervention.

Moreover, individuals with impaired safety of swallow, particularly from the community, demonstrated increased susceptibility to lower respiratory tract infections with notable odds ratios ranging from 2.39 to 2.55 (95% CI: 1.07–5.34 to 1.07–6.09, *p* < 0.05), emphasizing the association between swallowing difficulties and lower respiratory tract infections.

We observed influences on readmissions among older patients, especially from acute geriatric units, suggesting heightened odds for readmission, particularly for causes related to lower respiratory tract infections and other unrelated causes (odds ratios ranging from 1.37 to 5.97, 95% CI: 1.02–1.84 to 2.73–9.43, *p* < 0.05).

Additionally, post-stroke hospitalized patients with dysphagia exhibited a wide array of complications, including neurological complications, respiratory infections, urinary infections, and prolonged hospital stays. Odds ratios ranged from 2.11 to 27.34 (95% CI: 1.58–7.25 to 3.63–205.9, *p* < 0.001), signifying substantial associations between post-stroke complications and adverse outcomes.

## Discussion

Dysphagia is a complex condition that significantly impacts the health of diverse patient groups. Our review offers a comprehensive synthesis and examination of data compiled from five previously published studies conducted at Mataro Hospital. This meticulous examination aimed to consolidate and synthesize findings from these investigations, offering a robust evaluation of dysphagia and its associated complications in older and post-stroke patients. The examination of data from these studies highlighted significant patterns and relationships, enriching our comprehension of the substantial impact of dysphagia within diverse clinical contexts among these patient phenotypes.

Our review reaffirms insights from previous studies, emphasizing the increased risks associated with dysphagia in various patient groups. These conclusions echo documented associations between dysphagia and increased risks, consolidating existing knowledge within diverse patient cohorts. The rise in mortality, consistent with some prior studies, underscores the need for targeted interventions for dysphagia in pneumonia cases. The heightened risks of aspiration pneumonia, malnutrition, hospital readmissions, and mortality rates among individuals with dysphagia highlight the urgent need for increased awareness and targeted interventions. Our findings show a profound correlation between dysphagia and aspiration pneumonia, particularly among older individuals, where pneumonia onset significantly escalates mortality rates at both 1 month and 1 year. Aspiration pneumonia arises from compromised swallowing safety, declining immunity and frailty due to aging, and inadequate oral hygiene, fostering bacterial colonization in the respiratory tract (24). Older individuals with swallowing difficulty often exhibit compromised oral health, heightened colonization of respiratory pathogens, and an increased susceptibility to lower respiratory infections. Recent research, notably highlighted in the Japanese Respiratory Society Guidelines, emphasizes the need for thorough investigation and management strategies despite the ambiguous diagnostic criteria for aspiration pneumonia (24). Current studies outline common clinical parameters for diagnosing aspiration pneumonia, focusing on symptoms, inflammatory markers, and specific chest imaging patterns associated with aspiration. However, distinguishing aspiration pneumonia from non-aspiration pneumonia presents challenges, prompting a shift toward a comprehensive approach that prioritizes evaluating swallowing function prospectively, identifying causal factors, and exploring alternative diagnoses or dysphagia-related causes in the older population experiencing pneumonia. Our study underscores the urgency of early recognition and management of dysphagia to mitigate the significant risk of aspiration pneumonia, a crucial step in enhancing survival rates among this vulnerable demographic. A recent scoping review aimed to identify clinical competencies for managing aspiration pneumonia in older adults (25). Ninety-nine studies were analyzed, resulting in a refined list of 12 competencies covering diagnosis, treatment, support, and interdisciplinary collaboration, emphasizing a “Diagnose, Treat and SUPPORT” approach. These competencies urge healthcare professionals to collaborate, address unmet needs, and enhance patient care, particularly focusing on supportive care aspects.

Our review revealed a significant association between dysphagia and subsequent malnutrition risk in older hospitalized patients, underscoring the need for prompt evaluation and intervention. Dysphagia can result in malnutrition, leading to deficiencies in crucial nutrients like proteins, calories, and vitamins due to swallowing difficulties (23, 26, 27). Additionally, reduced fluid intake can cause dehydration, particularly hypovolemic dehydration, leading to health complications. The triple adaptation concept offers a comprehensive strategy to tackle these issues (28). It primarily involves adapting food texture and fluid viscosity to ensure safer swallowing practices. Secondly, it customizes caloric, protein, and hydration intake to target individual nutritional deficiencies. Lastly, it enhances sensory attributes to encourage adherence to dietary guidelines. The practical application of triple adaptation resulted in 296 diverse recipes across 16 weekly menus based on Mediterranean cuisine principles. These recipes cater to various textures, viscosities, nutritional needs, and seasonal variations, and enhance the organoleptic quality of the dishes (28). This diet adaptation, integrating scientific insights into practical dietary interventions, allows for the management of both dysphagia complexities and prevalent nutritional deficiencies, improving compliance and clinical outcomes.

Our findings confirm a significant correlation between dysphagia and hospital readmissions due to clinical complications, primarily respiratory infections. Specifically, patients with poorly managed dysphagia have a 1.82 times higher risk of pneumonia readmission and a 5.07 times higher risk of aspiration pneumonia readmission. While our study did not directly investigate economic implications, it highlights the link between dysphagia and these complex clinical complications, indicating potential healthcare cost implications. Previous studies have stressed the financial burden of managing such complications, highlighting the extensive impact of untreated or poorly managed dysphagia on healthcare costs. This correlation emphasizes the importance of early dysphagia identification and effective management in reducing these complications. Proactive dysphagia management could potentially prevent these challenging clinical outcomes, thereby enhancing patient care and reducing healthcare resource strain.

The lack of dysphagia awareness and knowledge among caregivers, including general practitioners, is clear in our studies and wider literature (14–18). This knowledge gap presents a significant challenge in recognizing and managing dysphagia. To address this, we have implemented an innovative solution that uses AI to prospectively assess the risk of dysphagia in every patient admitted to our institution. We developed an expert system based on machine learning, using electronic health records of all hospitalized older patients during admission. This expert system calculates the risk of dysphagia, providing an accurate and systematic screening process. A recent study involving 2,809 older patients demonstrated the expert system’s high predictive power, with a sensitivity of 0.940 and a positive predictive value of 0.834. The expert system efficiently screens all admitted patients in seconds, identifying those at greater risk of dysphagia and relaying this information directly to the clinician’s workstation in real time. Currently in active use at our institution, this AI-driven expert system offers a significant advancement in the early recognition of dysphagia, enabling the implementation of tailored diagnostic and therapeutic strategies for each patient (19). In response to the absence of evidence-based treatment protocols for dysphagia, we have introduced Minimal-Massive Intervention, a promising shift in dysphagia management (20). This innovative method, designed for hospitalized dysphagia patients and based on aspiration pneumonia’s pathophysiology, combines fluid modification, nutritional supplementation, and oral health recommendations (29). Our recent study demonstrated Minimal-Massive Intervention’s effectiveness, showing significant improvements in nutritional status and functionality, reduced hospital readmissions and respiratory infections, and increased 6-month survival rates. These findings position Minimal-Massive Intervention as a promising, cost-effective strategy to mitigate dysphagia complications. By providing caregivers with the necessary knowledge and skills, including Minimal-Massive Intervention awareness, we can optimize dysphagia care and reduce associated risks and costs. Optimal Massive Intervention is an evolution from Minimal-Massive Intervention, offering a more comprehensive and personalized approach to dysphagia management. While Minimal-Massive Intervention focuses on three basic elements to prevent dysphagia complications in older hospitalized patients, Optimal Massive Intervention incorporates multiple components targeting improved swallowing function and enhanced quality of life across various dysphagia causes. Optimal Massive Intervention requires a multidisciplinary team, care coordination, evidence-based interventions, and continuous outcome monitoring, aligning with current best practice guidelines for dysphagia management. Optimal Massive Intervention is currently being evaluated in a clinical trial to assess its effectiveness and cost-effectiveness compared to standard care (30).

Our study, while insightful, has certain limitations. Its retrospective nature restricts our ability to establish causal relationships and control all confounding variables, despite our thorough analysis of Mataró Hospital’s data. The study’s focus on a single, internationally recognized European center may limit the applicability of our findings to wider populations. The data, sourced from a single hospital with high standards of assessment, clinical management, and follow-up, may not reflect the practices of other hospitals or geographical locations. While our findings offer valuable insights into the factors causing illness in older adults with dysphagia at Mataró Hospital, multi-center studies are needed to validate these results in other settings. Lastly, our study serves as a synthesis of our previous findings, contributing to its narrative review nature. This approach, while providing a comprehensive overview of our work on dysphagia complications, lacks the robustness of prospective experimental designs or systematic reviews.

**Figure 1:**
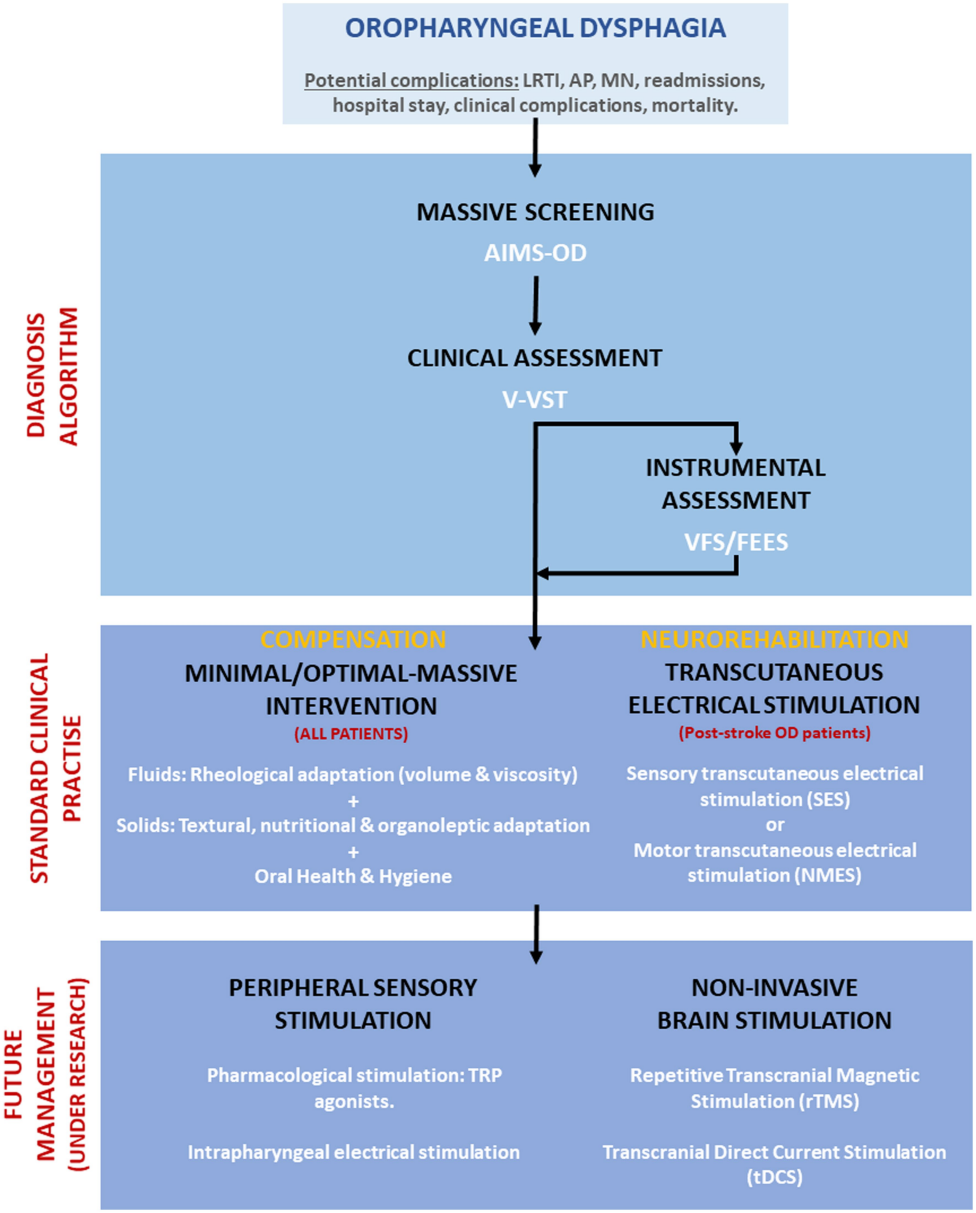
Algorithm for screening, diagnosis, and management of dysphagia at Hospital de Mataró. Future management treatments under research are also included. LRTI, Lower respiratory tract infections; AP, Aspiration pneumonia; MN, Malnutrition; AIMS-OD, Artificial intelligence massive screening for oropharyngeal dysphagia; V-VST, Volume-viscosity swallowing test; VFS, Videofluoroscopy; and FEES, Fiberoptic endoscopic evaluation of swallow.

After thorough problem identification and discussion, we have implemented a unique solution at our hospital that has shown promising results in addressing this issue. This is depicted in an algorithm (Figure 1) that showcases proactive universal screening, clinical diagnosis using volume-viscosity swallow test, and tailored management for dysphagia in older and post-stroke patients at Mataró hospital. Neurostimulation and transient receptor potential (TRP) stimulation are emerging as safe and effective interventions for dysphagia. Neurostimulation has been shown to enhance swallowing biomechanics, neurophysiology, induce cortical plasticity, and safety in people with dysphagia (31, 32). Electrical stimulation, including transcutaneous, neuromuscular, sensory, and pharyngeal, improves nerve or muscle function related to swallowing and has been shown to enhance swallowing safety, particularly in post-stroke patients with dysphagia (33–35). These techniques can enhance swallow response, reduce pharyngeal transit time, and lower penetration-aspiration scores. A low-intensity current treatment (25 mA, VitalStim device, FDA approved) endorsed by NICE guidelines (2018), is safe and prevents muscle damage and pain. However, it carries potential risks like muscle damage, infection, pain, and inconsistent effectiveness (36). High voltage stimulation may cause muscle damage if improperly administered (37). Non-invasive brain stimulation such as transcranial magnetic stimulation (TMS) and transcranial direct current stimulation (tDCS), can modulate the cortical activity related to swallowing by applying magnetic fields or direct currents to the scalp and can target specific brain regions involved in swallowing, such as the primary motor cortex, the premotor cortex, the supplementary motor area, and the insula (31, 38, 39). Non-invasive brain stimulation has been shown to enhance the swallowing function, reduce the aspiration risk, and increase the quality of life in people with dysphagia. Another promising treatment for dysphagia is the pharmacological stimulation of the TRP channels, which are expressed in the sensory nerves and epithelial cells of the oropharynx and larynx. TRP agonists, such as capsaicin, menthol, and piperine, can activate the TRPV1, TRPM8, and TRPV1/TRPA1 receptors, respectively, and modulate the sensory feedback and motor output of swallowing (31, 40). These treatments are well tolerated and do not cause major adverse events. Effectiveness of these treatments can vary significantly among individuals, influenced by factors like dysphagia’s severity and cause, the patient’s overall health, and treatment tolerance. Despite their potential demonstrated in clinical studies, further high-quality, large-scale randomized controlled trials are essential to confirm their efficacy and safety.

In conclusion, our review underscores the vital need for early recognition and intervention in dysphagia, particularly in older hospitalized patients and those with neurological complications. The studies we reviewed demonstrate that dysphagia is significantly linked with severe clinical complications like aspiration pneumonia in various phenotypes of older patients. The increased risks of aspiration pneumonia, malnutrition, hospital readmissions, and mortality rates among individuals with dysphagia highlight the urgent need for heightened awareness, systematic screening and diagnosis, and targeted interventions. It is essential to recognize dysphagia as a serious issue that warrants further attention and research.

## Data availability statement

The raw data supporting the conclusions of this article will be made available by the authors, without undue reservation.

## Ethics statement

Ethical approval was not required for the study involving humans in accordance with the local legislation and institutional requirements. Written informed consent to participate in this study was not required from the participants or the participants’ legal guardians/next of kin in accordance with the national legislation and the institutional requirements.

## Author contributions

TK: Conceptualization, Writing – original draft, Writing – review & editing. PC: Conceptualization, Writing – original draft, Writing – review & editing. OO: Conceptualization, Writing – original draft, Writing – review & editing.
